# Exogenous Melatonin Confers Cadmium Tolerance by Counterbalancing the Hydrogen Peroxide Homeostasis in Wheat Seedlings

**DOI:** 10.3390/molecules23040799

**Published:** 2018-03-30

**Authors:** Jun Ni, Qiaojian Wang, Faheem Afzal Shah, Wenbo Liu, Dongdong Wang, Shengwei Huang, Songling Fu, Lifang Wu

**Affiliations:** 1 Key Laboratory of High Magnetic Field and Ion Beam Physical Biology, Hefei Institutes of Physical Science, Chinese Academy of Sciences, Hefei 230031, Anhui, China; nijun@ipp.ac.cn (J.N.); liuwenbo9261@sina.com (W.L.); swhuang@ipp.ac.cn (S.H.); 2 School of Forestry and Landscape Architecture, Anhui Agricultural University, Hefei 230031, Anhui, China; wangqj521@126.com (Q.W.); faheemhorticulturist@gmail.com (F.A.S.); 15755059531@163.com (D.W.); fusongl001@outlook.com (S.F.)

**Keywords:** melatonin, wheat, cadmium stress, hydrogen peroxide, senescence

## Abstract

Melatonin has emerged as a research highlight regarding its important role in regulating plant growth and the adaptation to the environmental stresses. In this study, we investigated how melatonin prevented the cadmium toxicity to wheat seedlings. The results demonstrated that cadmium induced the expression of melatonin biosynthesis-related genes and cause a significant increase of endogenous melatonin level. Melatonin treatment drastically alleviated the cadmium toxicity, resulting in increased plant height, biomass accumulation, and root growth. Cadmium and senescence treatment significantly increased the endogenous level of hydrogen peroxide, which was strictly counterbalanced by melatonin. Furthermore, melatonin treatment caused a significant increase of GSH (reduced glutathione) content and the GSH/GSSG (oxidized glutathione) ratio. The activities of two key antioxidant enzymes, ascorbate peroxidase (APX) and superoxide dismutase (SOD), but not catalase (CAT) and peroxidase (POD), were specifically improved by melatonin. Additionally, melatonin not only promoted the primary root growth, but also drastically enhanced the capacity of the seedling roots to degrade the exogenous hydrogen peroxide. These results suggested that melatonin played a key role in maintaining the hydrogen peroxide homeostasis, via regulation of the antioxidant systems. Conclusively, this study revealed a crucial protective role of melatonin in the regulation of cadmium resistance in wheat.

## 1. Introduction

Stresses such as drought, cold, and salinity cause widespread crop losses throughout the world. The stresses could bring about a wide range of physiological, morphological, and anatomical disruptions in plants [1]. Although plants have developed a flexible induration to some certain abiotic stresses, this process is energy-costly, resulting in inhibited plant growth and decreased yield. Thus, it is important to develop stress-tolerant crops or methods to alleviate the adverse effects caused by stresses. Understanding the mechanisms of stress tolerance, along with identification of key genes and signal molecules involved in the stress signaling network, is pivotal in crop improvement.

Phytohormones, a diverse group of signaling molecules, are powerful regulators controlling plant growth and acclimation to the environmental stresses throughout the whole life cycle [2]. The stresses, such as drought, cold, and salinity, can alter the endogenous hormones level via direct or indirect regulation of the hormonal biosynthesis or degradation [3]. Although the plant response to stresses depends on many factors, phytohormones are considered as key endogenous molecules for modulating physiological and molecular responses [4]. Thus, phytohormones are potential targets for genetic engineering to improve the stress tolerance. Abscisic acid (ABA) is well known for conferring tolerance to abiotic stresses, such as drought and salinity [5]. Foliar application with low concentrations of ABA can improve the photosynthesis rate and biomass accumulation in *Brassica napus* growing under salinity conditions [6]. In many species, drought and salt stresses can also increase the level of salicylic acid (SA) and jasmonic acid (JA), which are closely associated with plant defense to biotic stresses [7,8]. Other hormones, such as auxins, cytokinins, gibberellins (GAs), and brassinolides (BRs), were also demonstrated to be involved in stress responses [2]. Strigolectones (SLs) were recognized as a new class of phytohormones involved in the regulation of shoot branching [9]. Recent studies have shown that they also positively regulated stress tolerance, such as to salt and drought [10].

Melatonin, which was originally discovered in the bovine pineal gland in 1958 [11], is a pleiotropic molecule with numerous cellular and physiological functions in animals [12]. The discovery of melatonin in higher plants demonstrated that melatonin was ubiquitous throughout the animal and plant kingdoms [13]. Subsequent studies revealed the important roles of melatonin in regulating plant growth, development, and responses to the various environmental stresses [14,15]. Melatonin acts as bio-stimulator in plants by enhancing the tolerance against abiotic stresses [16,17], including high temperature [18,19,20], cold and chilling [21,22,23,24,25], drought [26], salt [27,28,29], heavy metal [30,31,32,33,34], acid rain [35], and biotic stresses such as pathogen attacks [12]. Similar to the other abiotic stresses, cadmium pollution was a major environmental concern for its toxic actions to plants and animals [36]. Plants showed inhibited growth and decreased yield due to the serious morphological, metabolic, and physiological anomalies caused by cadmium [33]. Recent evidences also demonstrated that melatonin interacted with other phytohormones in the regulation of the responses to stresses. Melatonin treatment could increase the endogenous cytokinin (CK) level via up-regulation of two CK biosynthesis genes (*IP2* and *LOG1*) under heat stress in *Lolium perenne* [37]. Melatonin treatment also regulated the ABA or GA levels in response to salt, drought, and cold stress [38,39,40]. These results suggested that the regulation of the hormonal homoeostasis could contribute to the stress resistance conferred by melatonin.

Wheat, one of the most important crops in the world, is very sensitive to many environmental stresses, such as high temperature, salt, and heavy metals [41]. Cadmium pollution has great effects on the plant growth and yield of wheat, which has already been one of the most critical worldwide tasks. Several studies have reported that application with lime [42], proline [43], ABA [44], or SA [45] can promote the resistance to the cadmium stress by decreasing the cadmium uptake or toxicity in wheat. However, more research is still needed, with the aim to alleviate the cadmium toxicity or generate cadmium-resistant crops. In the present study, we focused on the effect of melatonin on the wheat seedlings under cadmium stress. The results demonstrated that cadmium promoted the endogenous melatonin biosynthesis in the wheat seedlings. Melatonin treatment promoted the resistance to cadmium stress and delayed the wheat leaf senescence. The present study may provide the physiological and biochemical basis for further investigation of the regulatory mechanism of melatonin-mediated tolerance to cadmium stress in wheat.

## 2. Results

### 2.1. Cadmium Treatment Promoted the Expression of Key Genes Involved in Melatonin Biosynthesis and Further Increased the Endogenous Melatonin Content in the Wheat Seedlings

**Figure 1:**
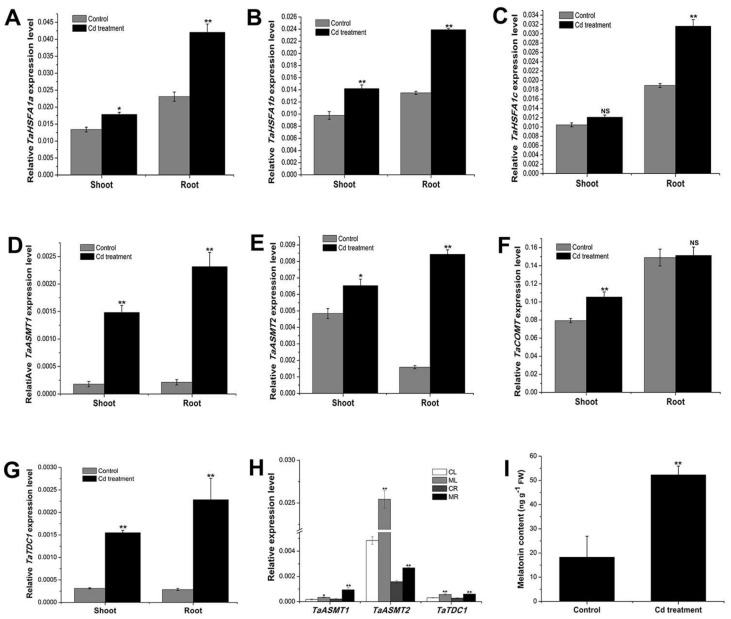
Cadmium treatment induced the expression of melatonin-biosynthesis-related genes and increased the melatonin level. Three-day-old wheat seedlings were root-treated with cadmium (0.2 mM CdCl_2_). The expression of *TaHSFA1a* (**A**), *TaHSFA1b* (**B**), *TaHSFA1c* (**C**), *TaASMT1* (**D**), *TaASMT2* (**E**), *TaCOMT* (**F**), and *TaTDC1* (**G**) were analyzed separately in the seedling shoot and root 24 h after treatment using qPCR (*n* = 3). *TaACT* was used as the internal reference. (**H**) Root treatment with melatonin (100 µM) induced the expression of *TaASMT1*, *TaASMT2*, and *TaTDC1* at 24 h (*n* = 3; CL, mock-treated leaf; ML, melatonin-treated leaf; CR, mock-treated root; MR, melatonin-treated root). (**I**) Melatonin content of the seedlings was analyzed 48 h after 0.2 mM Cd treatment (*n* = 3). Values are mean ± SE. Significance between treatment and control was determined by Student’s *t*-test. Significance level: * *p* < 0.05, ** *p* < 0.01, NS, no significance.

In plants, several genes, such as *N-acetylserotonin methyltransferase* (*ASMT*) [46], *Caffeic acid O-methyltransferase* (*COMT*) [47], *Tryptophan decarboxylase* (*TDC*) [48], and *Heat shock transcription factor A1* (*HSFA1*) [31] were involved in the regulation of melatonin biosynthesis. The homologs of these genes in wheat were identified (two *AMST* genes, one *COMT* gene, two *TDC* genes, and three *HSFA1* genes), and the detailed gene information is listed in Table S1. To investigate whether cadmium can affect endogenous melatonin homeostasis, the expression of melatonin biosynthesis genes and endogenous melatonin level were separately determined at 24 h and 48 h, after root treatment with cadmium (0. 2 mM). The results showed that, 24 h after cadmium treatment, the expression of all the melatonin biosynthesis genes was increased, both in the root and shoot of the seedlings (Figure 1A–G). It was also found that melatonin treatment increased the expression of several melatonin biosynthesis genes, such as *TaASMT1*, *TaASMT2*, and *TaTDC1* (the expression of other genes were not significantly changed, data not shown) in the shoot and root of the seedlings (Figure 1H), suggesting that melatonin has feedforward effects on its biosynthesis in wheat. Further, a significant increase of endogenous melatonin was detected at 48 h (Figure 1I), which were in accord with the increased expression of melatonin biosynthesis genes induced by cadmium stress. Taken together, these results suggested that cadmium and melatonin can trigger the biosynthesis of the endogenous melatonin in wheat, and the increased melatonin level could play a pivotal role in wheat seedlings coping with the cadmium stress.

### 2.2. Exogenous Melatonin Application Alleviated the Inhibition of Seedling Growth Induced by Cadmium Stress

**Figure 2:**
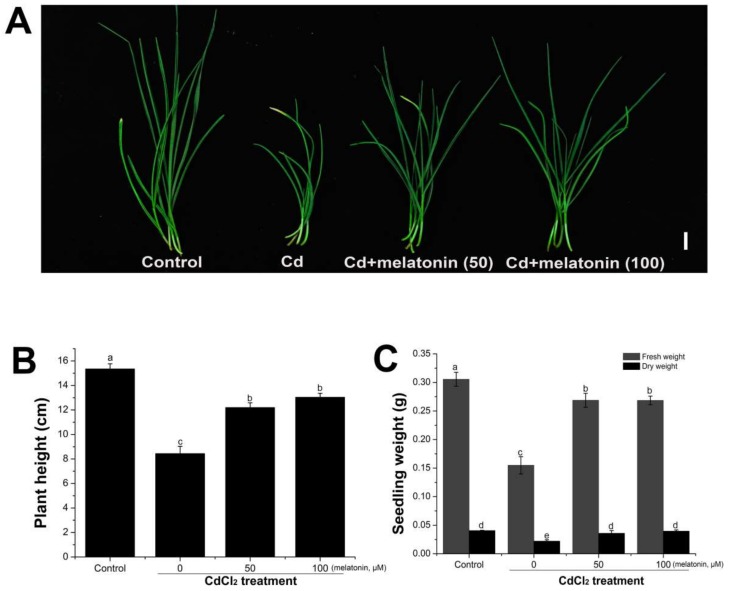
Melatonin alleviated the plant growth inhibition caused by cadmium treatment. (**A**) Seedling growth one week after CdCl_2_ (0.2 mM), or CdCl_2_ (0.2 mM) + melatonin (50 and 200 µM) treatment. (**B**,**C**) Plant height and shoot weight were calculated one week after cadmium (0.2 mM) or cadmium + melatonin treatment (*n* = 20). Values are mean ± SE. Means with different letters (a, b, c, d and e) are significantly different at *p* < 0.05 using Tukey’s test. Bars = 1 cm.

The observation of a marked increase of the endogenous melatonin in wheat seedlings after cadmium treatment suggested that melatonin could be involved in some physiological functions of the responses to cadmium stress. Firstly, the effects of different concentrations of cadmium on the growth of the wheat seedlings were investigated. The plant growth and biomass accumulation were dramatically decreased with increasing cadmium concentrations (Figure S1). To investigate whether melatonin can alleviate the inhibition caused by cadmium stress, melatonin (0, 50, and 100 µM) was co-applied with cadmium (0.2 mM) to the root of the four-day-old wheat seedlings. One week after treatment, it was found that melatonin dramatically alleviated the inhibition of plant growth induced by cadmium treatment (Figure 2). Compared with the cadmium-treated group, the plant height (Figure 2A,B) and fresh and dry weight (Figure 2C) of the melatonin-co-treated plants was significantly increased. It has been reported that melatonin treatment can enhance the plant growth in soy bean [49] and *Malus rockii* [50]. These results demonstrated that melatonin was directly involved in the regulation of plant responses to the cadmium stress, while exogenous melatonin treatment alleviated the growth inhibition of the wheat seedlings under cadmium stress.

### 2.3. Melatonin Affected the Primary Root Elongation and Alleviated the Root Growth Inhibition Caused by Cadmium Stress

**Figure 3:**
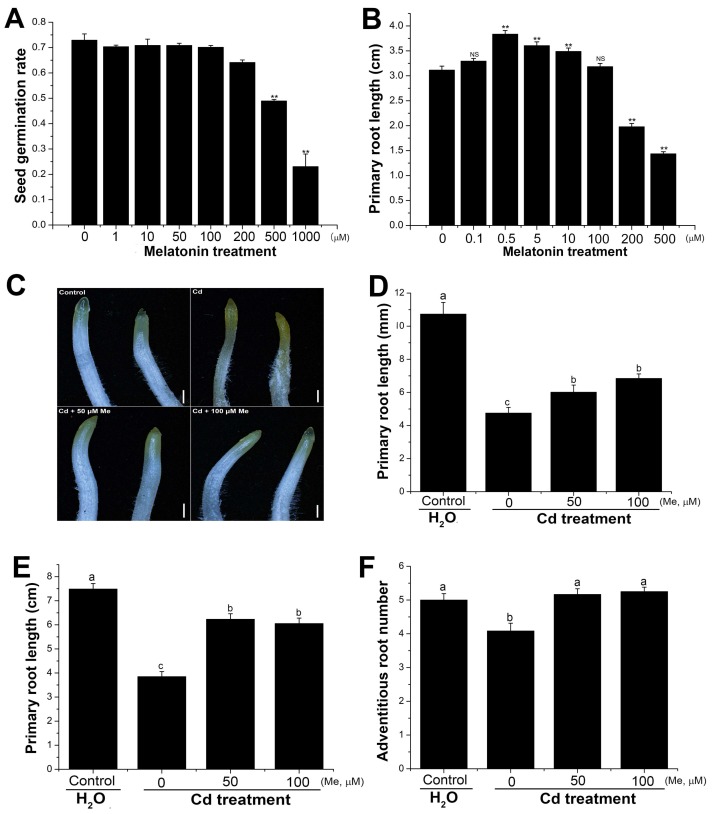
Melatonin promoted the primary root elongation and alleviated the toxicity of cadmium to the root growth on the wheat seedlings. (**A**) Effect of different concentrations of melatonin on the seed germination at 24 h after seed priming (*n* = 4). (**B**) Primary root length at 2 d after root treatment with different concentrations of melatonin (*n* = 20). (**C**,**D**) Root growth of the germinated seedlings at 24 h after cadmium (0.2 mM), or cadmium + melatonin treatment (*n* = 20). (**E**,**F**) Primary root length and adventitious root number were calculated three days after treatment (*n* = 20). Values are mean ± SE. Means with different letters (a, b and c) are significantly different at *p* < 0.05 using Tukey’s test. Significance between treatment and control was determined by Student’s *t* test. Significance level: ** *p* < 0.01. NS, no significance.

Environmental stresses, such as drought, salt, and heat stresses, could directly affect the seed germination. It has been reported that cadmium treatment inhibited the seed germination in *Brassica napus* [51], *Achnatherum inebrians* [52], and *Sorghum bicolor* [53]. In this study, the results showed that the wheat seed germination was highly tolerant to cadmium stress. The inhibition of seed germination can be detected, only at very high concentrations of CdCl_2_ (e.g., 4 mM) (Figure S2). Intriguingly, low concentrations of CdCl_2_ (e.g., 0.5 mM) promoted the wheat seed germination (Figure S2). In *Arabidopsis thaliana*, melatonin treatment significantly promoted the seed germination. This is contrary to wheat, where seed priming with melatonin of low concentrations had no significant effects on the seed germination (Figure 3A).

In rice, melatonin had inhibitory effects on the primary root growth [54]. As comparison, different concentrations of melatonin were applied to the germinated wheat seeds. The results showed that melatonin (0.5, 5, and 10 µM) promoted the elongation of the primary root (Figure 3B), the growth of which was inhibited, only by high concentrations (e.g., 200 µM melatonin), suggesting that unlike in rice, melatonin was positively regulated the primary root growth in wheat. Contrary to this, cadmium showed inhibitory effects on the root tip and primary root elongation. As shown in Figure 3C, cadmium treatment (0.2 mM) stressed the shoot tip and promoted the pigment accumulation at 12 h after treatment, revealing a fast and strong stress response to cadmium in the root. Likewise, the primary root growth was significantly inhibited (Figure 3D). The average root length and the adventitious root number were decreased by cadmium treatment (0.2 mM) at 3 d after germination (Figure 3E,F). However, co-application of melatonin (50 and 100 µM) drastically decreased the stress-induced pigment accumulation in the root tip, promoted the primary root elongation, and increased the adventitious root number (Figure 3C–F). These results suggested that melatonin plays a protective role in maintaining the root architecture under cadmium stress.

### 2.4. Melatonin Counterbalanced the Endogenous Hydrogen Peroxide Level Induced by Cadmium Stress and Prevented the Toxicity to the Root from the Exogenous Hydrogen Peroxide

**Figure 4:**
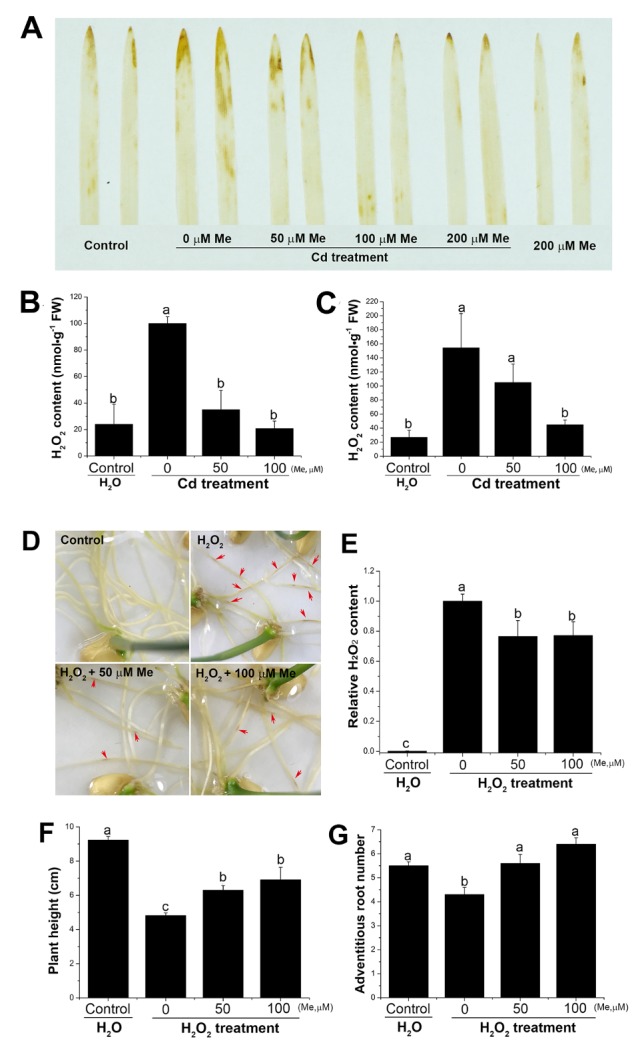
Melatonin decreased the hydrogen peroxide level induced by cadmium stress and prevented the toxicity of exogenous hydrogen peroxide to the root. (**A**) In situ detection of hydrogen peroxide after cadmium (0.2 mM), melatonin, or cadmium + melatonin treatment. (**B**,**C**) Hydrogen peroxide content was analyzed at 2 d after cadmium (0.2 mM), cadmium + melatonin treatments, separately in the shoot and root (*n* = 5). (**D**) Root growth at 2 d after 0.3% H_2_O_2_, or 0.3% H_2_O_2_ + melatonin treatment. (**E**) Relative H_2_O_2_ content in the culture medium at 2 d after treatment (*n* = 3). (**F**,**G**) Plant height and adventitious root number were separately calculated at 5 d after treatment (*n* = 20). For determination of plant fresh and dry weight, three seedlings were mixed as one sample. Values are mean ± SE. Means with different letters (a, b and c) are significantly different at *p* < 0.05 using Tukey’s test. Bars = 1 cm.

Cadmium stress inhibited the plant growth via increasing the oxidative chemicals, mainly hydrogen peroxide, of which the over-dosed accumulation in the plant cells will lead to the subsequent oxidative stresses [55]. In the wheat seedlings, the results showed that root application with cadmium (0.2 µM) significantly elevated the endogenous hydrogen peroxide level, both in roots and shoots (Figure 4). Nevertheless, the hydrogen peroxide level was decreased, with the existence of melatonin, which was determined by in situ histochemical staining (Figure 4A) and hydrogen peroxide quantification (Figure 4B,C). These results suggested that melatonin acted as a hydrogen peroxide scavenger in counteracting the accumulation of hydrogen peroxide induced by cadmium stress in the wheat seedlings. To further validate the effects of hydrogen peroxide on the growth of wheat seedlings, low concentrations of hydrogen peroxide (0.3%) were directly applied to the seedling root. Exogenous application with hydrogen peroxide caused significant inhibition on the seedling growth, resulting in inhibited root growth (Figure S3A), decreased plant height, chlorophyll content, and biomass accumulation (Figure S3B–D), which is similar to the growth inhibition after cadmium treatment (Figure 2). Interestingly, pretreatment with melatonin significantly alleviated the toxicity of the exogenous hydrogen peroxide to the root (Figure 4D). Additionally, hydrogen peroxide content in the medium was decreased by melatonin treatment (Figure 4E), suggesting that melatonin could improve the ability of the seedling root to degrade the exogenous hydrogen peroxide. The plant height and adventitious root number were also increased when co-applied with melatonin (Figure 4F,G). Conclusively, these results demonstrated that the increased hydrogen peroxide level in the shoot and root of the wheat seedlings could be one of the main reasons for the growth inhibition caused by cadmium treatment, where melatonin can alleviate the inhibitory effects via counter-balancing the endogenous hydrogen peroxide level.

### 2.5. Melatonin Delayed the Leaf Senescence via Preventing the Hydrogen Peroxide Accumulation

**Figure 5:**
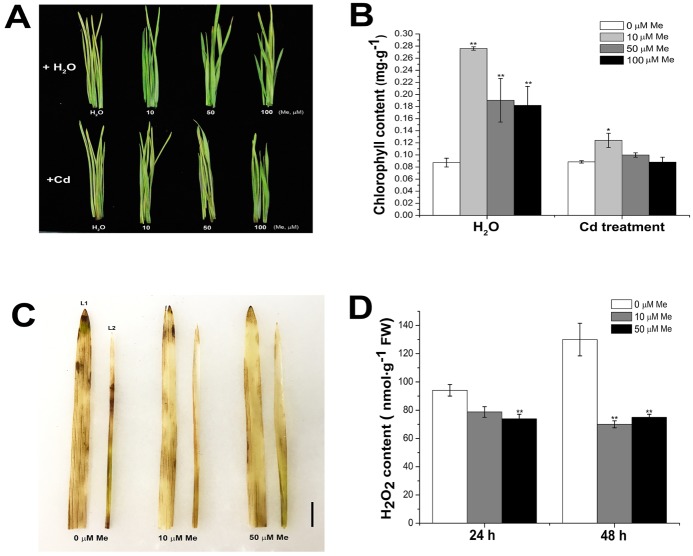
Melatonin delayed the senescence of the detached wheat leaves. (**A**) Photograph of representative leaves in response to senescence treatment after melatonin and CdCl_2_ (0.2 mM) + melatonin treatment. (**B**) Chlorophyll content upon senescence treatment at 10 d (*n* = 5). (**C**,**D**) In situ detection and quantification of the endogenous hydrogen peroxide at 24 and 48 h after senescence treatment (*n* = 5). Values are mean ± SE. L1 and L2 indicated the first and second leaf of the wheat seedlings. Bars = 1 cm. Significance between treatment and control was determined by Student’s *t* test. Significance level: * *p* < 0.05, ** *p* < 0.01.

Leaf senescence is a programmed cell death process, the initiation of which is controlled by many eternal and environmental factors. Delay of the leaf senescence in the crops could extend the photosynthesis duration and the nutrients relocation to the sink tissues [56]. In the wheat seedlings, cadmium treatment not only showed direct inhibition on the plant growth (Figure S1), but also induced the leaf senescence (Figure S4). We further investigated the effects of melatonin on the wheat leaf senescence, which was mimicked by leaf detachment. The results also showed that senescence treatment induced the expression of most melatonin biosynthesis genes in the wheat seedlings (Figure S5). To further investigate whether melatonin can delay the leaf senescence in the wheat seedlings with or without cadmium stress, the detached leaves were incubated with melatonin (10, 50, and 100 µM) or water for ten days at 25 °C, under a 16-h light/8-h dark cycle. The results showed that melatonin-treated wheat leaves had higher chlorophyll content than controls, with or without cadmium (0.2 mM) (Figure 5A,B), suggesting that melatonin treatment delayed the leaf senescence in the wheat seedlings. The production of reactive oxygen species (ROS), mainly hydrogen peroxide, has been regarded as the initial signal inducing leaf senescence [57]. In situ detection and quantitative determination of hydrogen peroxide in the detached leaves showed that senescence treatment significantly increased the endogenous hydrogen peroxide level (Figure 5C,D). However, melatonin application significantly decreased the hydrogen peroxide content in the detached leaves, suggesting that melatonin conferred the tolerance to leaf senescence, probably via counteracting the endogenous accumulation of hydrogen peroxide.

### 2.6. Melatonin Improved the Antioxidant Activities Under Cadmium Stress

**Figure 6:**
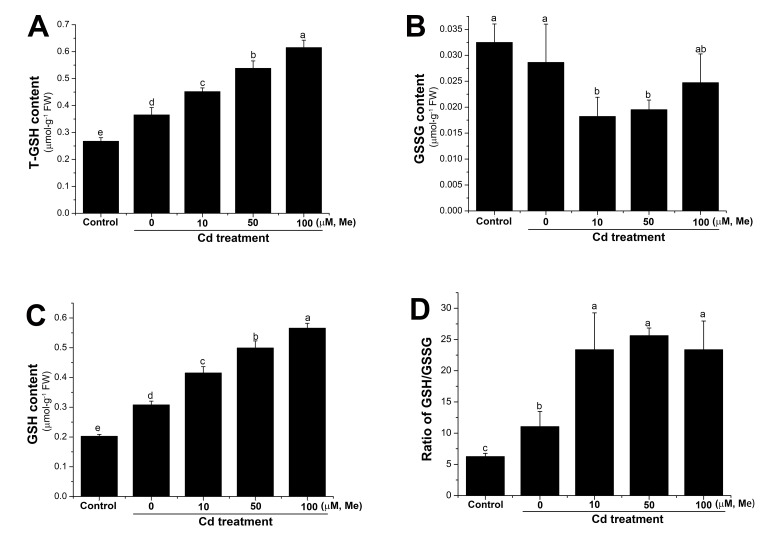
Glutathione homeostasis in wheat seedlings after cadmium, or cadmium + melatonin treatments. The total glutathione (**A**), oxidized glutathione (**B**), reduced glutathione (**C**) and GSH/GSSG ratio (**D**) of the four-day-old wheat seedlings were determined at 12 h after root treatment with cadmium (0.2 mM), or melatonin + cadmium. GSH content = T-GSH − 2GSSG. Data are means ± SE of three replicates. Means with different letters (a, b, c, d and e) are significantly different at *p* < 0.05 using Tukey’s test. Me, melatonin; GSH, reduced glutathione; GSSG, oxidized glutathione; T-GSH, total glutathione.

As shown in Figure 4, cadmium stress can cause fast and significant hydrogen peroxide accumulation in the wheat seedlings. As melatonin itself was unable to directly scavenge the hydrogen peroxide [58], then the regulation of hydrogen peroxide homeostasis by melatonin could be due to its ability to trigger the antioxidant systems. Thus, we further investigated how the antioxidant systems, including enzymatic and non-enzymatic systems, responded to melatonin under cadmium stress. It has been reported that elevated GSH (reduced glutathione) level is closely correlated with the abiotic stress tolerance, for GSH can directly scavenge the superoxide and hydroxyl radicals [59]. The GSH/GSSG (oxidized glutathione) ratio, in some distance, reflected the intensity of oxidative stress [23]. In this study, the GSH and GSSG level of the wheat seedlings was investigated at 12 h after cadmium (0.2 mM), or cadmium (0.2 mM) + melatonin (0, 10, 50, and 100 µM) treatment. The results showed that cadmium could increase the GSH content in the wheat seedlings (Figure 6). Nevertheless, melatonin caused a further increase of GSH level and higher GSH/GSSG ratio (Figure 6). These results suggested that GSH, which is the representative of non-enzymatic antioxidant systems, was positively regulated by cadmium stress, and the level of which can be further improved by melatonin in the wheat seedlings. The decrease of the endogenous hydrogen peroxide in the wheat seedlings might be closely correlated with the elevated GSH level after melatonin treatment.

**Figure 7:**
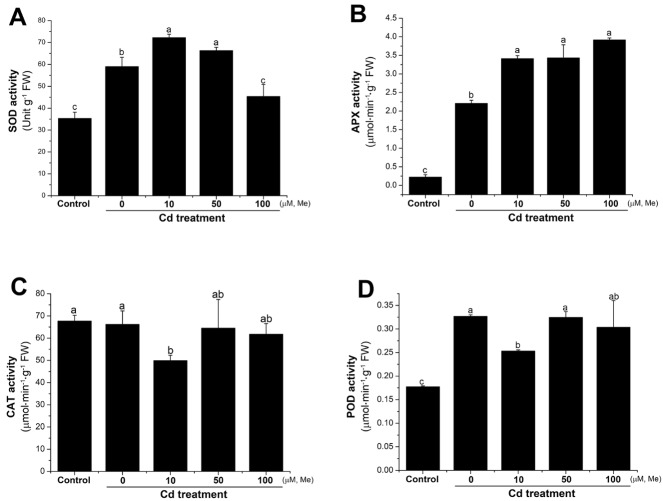
Melatonin affected the activities of key antioxidant enzymes in response to cadmium stress. The activities of SOD (**A**), APX (**B**), CAT (**C**), and POD (**D**) of the four-day-old wheat seedlings were separately determined at 12 h after root treatment with cadmium (0.2 mM), or melatonin + cadmium. Data are means ± SE of three replicates. Means with different letters (a, b, and c) are significantly different at *p* < 0.05 using Tukey’s test. Me, melatonin; SOD, superoxide dismutase; APX, ascorbate peroxidase; CAT, catalase; POD, peroxidase.

Various antioxidant enzymes are also considered as major antioxidants, in response to the environmental stresses. Superoxide dismutase (SOD) is the key enzyme that catalyzes the disproportionation of O_2_^−^ into H_2_O_2_, and then H_2_O_2_ can be further catalyzed into H_2_O by ascorbate peroxidase (APX), catalase (CAT), or peroxidase (POD) [60]. To investigate the role of melatonin in the regulation of the antioxidant enzymes, the activities of SOD, APX, POD, and CAT were separately assayed at 12 h after root treatment with cadmium (0.2 mM), or cadmium (0.2 mM) + melatonin (0, 10, 50, and 100 µM). The results showed that cadmium significantly increased the activity of SOD, APX, and POD, but not CAT (Figure 7). Interestingly, the activities of APX and SOD were further improved by melatonin under cadmium stress (Figure 7A,B). These results suggested that SOD, APX, and POD are cadmium-responsive antioxidant enzymes, and the activity of SOD and APX can be specifically up-regulated by melatonin. Conclusively, these results provided evidence that the alleviation of oxidative stress by melatonin on the wheat seedlings under cadmium stress was closely associated with the increased activities of the antioxidant systems in the wheat seedlings.

## 3. Discussion

In recent years, a number of studies have revealed the role of melatonin in plants as a protector against abiotic and biotic stresses [61]. Melatonin can alleviate the abiotic stresses caused by chilling, cold, salt, and heat in many species. However, only a few studies demonstrated the protective role of melatonin in regulating plant growth under cadmium stress. In tomato, alfalfa, and rice, melatonin was found to confer cadmium tolerance in the seedlings [31,32,33]. In wheat, the biological functions of melatonin on the regulation of plant growth and resistance to stresses still largely remain unclear. In the present study, the potential role of melatonin in alleviating the cadmium toxicity in the wheat seedlings was investigated.

In rice, the level of endogenous melatonin was increased by cadmium treatment via regulating the expression of *tryptophan decarboxylase* (*TDC*), *tryptamine 5-hydroxylase* (*T5H*), and *N-acetylserotonin methytransferase* (*ASMT*) [62]. In accord with these findings, our present results showed that cadmium stress also significantly induced the expression of the key genes regulating melatonin biosynthesis in wheat. The expression level of *HSFA1*, *ASMT*, *COMT*, and *TDC1* in both shoot and root of the wheat seedlings was significantly up-regulated 24 h after root treatment with cadmium, and cadmium treatment further increased the endogenous melatonin level (Figure 1). These results demonstrated that melatonin could be involved in the responses to cadmium stresses in wheat seedlings. Notably, melatonin itself can also rapidly induce the expression of *ASMT1* and *TDC1* (Figure 1H). The self-activation of melatonin biosynthesis could be a very effective strategy for fast accumulation of endogenous melatonin in wheat when coping with the environmental stresses.

The excessive production of reactive oxygen species, such as hydrogen peroxide, is one of the main reasons of plant growth inhibition under the abiotic stresses. A wild range of plant responses can be triggered by hydrogen peroxide, such as acclimation to the environmental stresses and induction of senescence [57]. Thus, it seems very important to maintain the endogenous hydrogen peroxide in a restricted level. In pea, there are evidences proving that cadmium could induce the antioxidant responses in all plant organs and increase the level of hydrogen peroxide, which is a direct agent of oxidative stress. In wheat, it has been reported that cadmium can induce the hydrogen peroxide production in the wheat root, but not in the leaf [63]. However, our results showed that the hydrogen peroxide level, both in the shoot and root, was increased after root treatment with cadmium (Figure 4). Co-application with melatonin drastically decreased the hydrogen peroxide content, confirming the common role of melatonin acting as ROS scavenger in plants. Further, the effects of exogenous hydrogen peroxide on the growth of the wheat seedlings were also evaluated. Root treatment with low concentrations of hydrogen peroxide inhibited the plant growth, reduced the chlorophyll content, and induced the leaf senescence (Figure S3). Nevertheless, it was worthy to mention here that melatonin not only prevented the toxicity from the exogenous hydrogen peroxide, but also improved the ability of the wheat root to degrade the exogenous hydrogen peroxide (Figure 4D,E), which is direct evidence revealing that melatonin was indeed involved in controlling the endogenous hydrogen peroxide homeostasis. Similarly, in some species, melatonin can delay the leaf senescence either by exogenous melatonin treatment or overexpressing melatonin biosynthesis gene *SNAT1* [64,65]. Previous studies suggested that the stress-induced leaf senescence is mainly due to the increased hydrogen peroxide level in the leaf [27]. Our results also confirmed that the endogenous hydrogen peroxide level in wheat leaves was indeed increased by senescence treatment (Figure 5). Similar to that under cadmium stress, melatonin reduced the endogenous hydrogen peroxide level and increased the chlorophyll content of the detached leaves (Figure 5). Taken together, these results suggested that melatonin is functionally involved in alleviating cadmium stress and delaying leaf senescence, probably via regulation of the endogenous hydrogen peroxide level.

Melatonin itself can not directly scavenge the oxidative radicals [58], thus the scavenge of the ROS could depend on the immediate activation of the antioxidant systems. GSH is the major non-enzymatic antioxidant, due to its pivotal role playing in the reduction of H_2_O_2_ to H_2_O. Acting as a cellular redox buffer, the GSH pool is essential for maintaining the ROS homeostasis when oxidative stress occurs [66]. It has been reported that environmental stresses can elevate the GSH level [59], demonstrating that GSH biosynthesis is an intrinsic plant response to stresses. In this study, cadmium treatment increased the GSH content and GSH/GSSG ratio (Figure 6). Co-application with melatonin resulted in a high beneficial value of GSH/GSSG ratio (Figure 6), which could strengthen the ability of the plant to deal with the oxidative stresses, in comparison with control and Cd treatment. These results suggested that the stimulation of GSH biosynthesis might be very important for melatonin in counterbalancing the redox homeostasis. Recent research also showed that melatonin could affect the activity of several antioxidant enzymes in plants. Exogenous application with melatonin increased CAT and POD activities in apple leaves under drought stress [67]. In wheat seedlings, the activities of SOD, Glutathione Peroxidase (GPX), APX, and Glutathione Reductase (GR), but not CAT, were increased by melatonin treatment under chilling stress [68]. In cucumber, melatonin treatment also increased the activities of SOD, POX, and GSSG-R, but not CAT [23], suggesting that CAT might be not responsive to melatonin treatment. SOD is the key enzyme that catalyzes the disproportionation of O_2_^−^ into H_2_O_2_, and then H_2_O_2_ can be further catalyzed into H_2_O by APX [60]. In this study in wheat seedlings, we found that SOD, APX, and POD, but not CAT, are responsive to cadmium stress. Exogenous treatment with melatonin further improved the activities of APX and SOD, but not POD and CAT (Figure 7). These results suggested that in wheat, SOD, APX, and POD, but not CAT, are cadmium-responsive antioxidant enzymes, and further, melatonin could specifically improve the activity of SOD and APX in response to cadmium stress. Conclusively, these results suggested that melatonin can improve the redox homeostasis by triggering the non-enzymatic and enzymatic antioxidant systems to protect from cadmium-induced oxidative stress.

In plants, both melatonin and auxins are indole-compounds and share a common biosynthesis pathway using tryptophan as the precursor, thus it was postulated that melatonin may have auxin-like functions in the regulation of plant growth [61]. In some monocot plants, melatonin acts as auxin that can promote the growth of shoots, but inhibit the embryonic root growth [69]. In rice and *Arabidopsis*, melatonin seemed to inhibit the embryonic root growth in an auxin-signal-pathway-dependent manner [54]. It has been demonstrated that melatonin is beneficial to plants by increasing root branching, but the signaling pathway was very likely independent of auxin responses [70]. In wheat, we showed here that the primary root growth was drastically inhibited by indole butyric acid (IBA) treatment (Figure S6), whereas it was promoted by low concentrations of melatonin (Figure 3B), suggesting that melatonin and auxin had divergent functions in regulating wheat root growth. The effects of auxin on the plant responses to cadmium stress were also investigated, in comparison with melatonin. Unlike that by melatonin treatment, auxin only slightly increased the biomass accumulation (Figure S7). These results suggested that, in wheat, melatonin does not act like auxin, at least in the regulation of root growth and the responses to cadmium stresses.

In conclusion, the results of this study demonstrated that melatonin strictly counterbalanced the endogenous hydrogen peroxide that induced by cadmium stress, via regulating the non-enzymatic and enzymatic antioxidant systems in the wheat seedlings. Melatonin positively regulated the primary root growth and improved the capacity of the root in the detoxification of cadmium and hydrogen peroxide. Together with the recent findings in wheat, melatonin can be regarded as a key protector from many environmental stresses and also showed its great potential for the practical applications on wheat cultivation. The future work will be focused on the identification and characterization of the molecular networks of melatonin in the regulation of abiotic stresses in wheat.

## 4. Materials and Methods

### 4.1. Plant Materials and Growth Conditions

Seeds of wheat (*Triticum aestivum* cv. Lianmai 7) were washed with distilled water twice and then primed in the distilled water for 12 h. Then, the seeds were placed on the pre-wetted filter paper. Approximately 6–12 h later, the germinated seeds were selected and planted on the pre-wetted five-layer filter paper (18 cm) in the petri dishes, and then covered with a transparent lid. Plants were grown at 25 °C, 100 µmol·m^2^·s^−1^ radiation, using a 14-h light/10-h dark cycle in a growth chamber. An amount of 20 mL distilled water was added into the petri dishes the second day after seed plantation. The lids of the petri dishes were removed two days after seed germination, when the seedlings were approximately 2–3 cm high. The seedlings were watered twice one day with approximately 50 mL water for one petri dish.

### 4.2. Cadmium, Melatonin, Indole Butyric Acid (IBA), and H_2_O_2_ Treatment

To make stock solutions (200 mM for CdCl_2_, 50 mM for melatonin, and 20 mM for IBA), CdCl_2_ (Sinopharm, Guoyao, China) was dissolved in distilled water, melatonin (Sigma, Ronkonkoma, NY, USA) in ethanol, and IBA (Sigma) in 0.2 mM NaOH solution. The stock solutions were used to prepare the working solutions of different concentrations. For H_2_O_2_ treatment, the stock solution (30%) was directly diluted with distilled water into different concentrations of working solutions. All working solutions for each treatment had the same solvents. For CdCl_2_, melatonin, CdCl_2_ + melatonin, IBA, or H_2_O_2_ treatment, 50 mL working solutions were prepared and directly applied to the root of seedlings growing in the Petri dish.

### 4.3. Seed Germination Test

After being primed in the distilled water, cadmium, melatonin, or H_2_O_2_ for 12 h, the seeds were then placed in the 9-cm-diameter Petri dishes with three layers of pre-wetted filter paper at 25 °C in darkness. The germination test was performed on samples of 450 seeds (150 seeds per dish, three biological replicates). Seeds with visible white radicle after the seed coat was broken were scored as germinated.

### 4.4. Melatonin Determination in the Wheat Seedlings

Two days after cadmium treatment on the three-day-old wheat seedlings, the samples were collected and frozen in liquid nitrogen. Three biological replicates for each treatment were pooled from 20 seedlings growing in one Petri dish. The samples were kept at −80 °C before use. The melatonin content was determined by high performance liquid chromatography (HPLC) as previously reported [71]. After ground in the liquid nitrogen, the samples (approximately 0.2 g) were added in the 2 mL chloroform. After being vortexed at maximum speed for 1 min, the samples were kept at 4 °C in the dark overnight. The samples were centrifuged at 13,000× *g* at 4 °C for 15 min. A 200 µL chloroform extract was evaporated to dryness and then dissolved in 0.1 mL 42% MeOH. Aliquots (10 µL) were analyzed in an HPLC system equipped with a fluorescence detector system (2475, Waters, Milford, MA, USA). The samples were separated on a Sunfire C18 column (4.6 × 150 mm, Waters). Melatonin was detected at 280 nm (excitation) and 348 nm (emission) as previously described [46].

### 4.5. Senescence Treatment

The senescence treatments were conducted as previously reported [32]. The leaves of five-day-old wheat seedling leaves were detached for the leaf senescence experiment. The detached leaves were transferred to 50-mL polypropylene tubes containing 20 mL distilled water or different concentrations of CdCl_2_, CdCl_2_ + melatonin, or melatonin solutions and incubated at 25 °C under 16-h light/8-h dark conditions. Ten days after incubation, the wheat leaves were used for determination of total chlorophyll content. Chlorophyll isolation was also followed as reported [32]

### 4.6. RNA Isolation and Quantitative Real Time PCR (qPCR)

The total RNA from the wheat shoot and root samples was isolated by using a Plant Total RNA Isolation Kit (Omega, Shanghai, China). The RNA quality and quantity were separately determined by gel electrophoresis and Scandrop spectrophotometer (Analytikjena, Jena, Germany). To avoid DNA contamination, the RNA was used as the template for PCR to amplify the *TaACTIN2* gene. First-strand cDNA was synthesized from 1–1.5 mg total RNA using PrimeScript™ RT reagent kit (TaKaRa biotechnology, Dalian, China). The quality of the cDNA was generally determined by PCR amplification of reference gene *TaACTIN2* (25 cycles). After electrophoresis, a bright band sometimes indicated the cDNA is qualified for the following qPCR experiment. Before qPCR, the quality of the qPCR primers was evaluated by RT-PCR to check whether the primers were good enough for target gene synthesis and the product is unique. The qPCR was carried out following the instruction of QuantiNova Multiplex PCR Kit (Qiagen, Shanghai, China) on a Lighter Cycler 480 qPCR machine (Roche, Basel, Swiss). The qPCR program was set as follows: preheating: 95 °C 10 min, one cycle; Amplification: 95 °C 10 s, 60 °C 20 s, and 72 °C 20 s, 45 cycles; Melting curve: 95 °C, 2 min, 60 °C, 30 s, then continuously increased to 95 °C. The primer sequences are listed in Table S1.

### 4.7. Determination and Histochemical Staining of Hydrogen Peroxide

DAB staining was used for the in situ detection of the endogenous hydrogen peroxide according to the previously described method [30]. The detached wheat leaves were submerged in the 3,3′-diaminobenzidine (DAB) solution (1 mg·mL^−1^, pH 3.8). After being incubated overnight at room temperature, the wheat leaves were then submerged in the water-free ethanol for 6–12 h to wash off the chlorophyll. For hydrogen peroxide determination, the shoot or root of the wheat seedlings were separately collected and stored in −80 °C. The hydrogen peroxide content of the samples was determined with little modification according to [23]. Fresh tissues were ground in a mortar with a pestle using liquid nitrogen. The sample powder (approximately 0.1–0.2 g) was transferred to the weighted 2 mL EP tube containing 1 mL 0.1% trichloroacetic acid (TCA). The EP tube was weighed again to calculate the sample weight. After that, the EP tube was vortexed at maximum speed for two minutes. The homogenate was centrifuged at 13,000× *g* for 15 min at 4 °C. The supernatant (0.5 mL) was added to the reaction medium containing 0.5 mL 10 mM phosphate (K) buffer and 1 mL 1 M potassium iodide (KI). The mixture was vortexed at maximum speed for one minute. The absorbance was determined at 390 nm by a Scandrop spectrophotometer (Analytikjena). The hydrogen peroxide content was calculated according to the hydrogen peroxide standard curve.

### 4.8. GSH Determination

The total GSH and GSSG content were determined by using Glutathione Pool Estimation Kit (Jiancheng Bioengineering Institute, Nanjing, China). The wheat samples (0.2 g FW) were powdered in the liquid nitrogen. According to the kit instructions, the GSH-T and GSSG content were determined based on the changes in absorbance at 412 nm (A_0.5min_ and A_10.5min_). GSH was estimated as the differences between the amount of GSH-T and GSSG as following: GSH = GSH-T − 2GSSG. The concentrations of GSH-T and GSSG were calculated according to the GSH and GSSG standard curve. The results were represented as µmol per gram fresh weight (µmol·g^−1^ FW).

### 4.9. Antioxidant Enzyme Extraction and Activity Assays

The antioxidant enzyme activities were determined by using spectrophotometric method. For extraction of enzymes, the seedling samples were ground into powder in the liquid nitrogen, then suspended in the ice-cold phosphate buffer (0.1 M, pH = 7). The homogenates were vortexed for 1 min, then centrifuged at 4 °C for 15 min at 12,000 rpm. The supernatants were used for the determination of enzymatic activity. The superoxide dismutase (SOD) activity was determined according to the instructions of Total Superoxide Dismutase (T-SOD) assay kit (Jiancheng Bioengineering Institute). One unit of SOD activity was defined as the amount of enzyme required to cause a 50% inhibition of the reduction rate monitored at 550 nm. The ascorbate peroxidase (APX) activity was determined according to the Ascorbate Peroxidase (APX) test kit (Jiancheng Bioengineering Institute). The APX activity was determined based on the changes in absorbance at 290 nm (A_10s_ and A_130s_). The catalase (CAT) and peroxidase (POD) activities were determined by using Catalase (CAT) Assay Kit and Peroxidase Assay Kit, separately (Jiancheng Bioengineering Institute).

### 4.10. Statistical Analysis

The data presented were analyzed via one-way analysis of variance (ANOVA), followed by Tukey’s test. A *p*-value of <0.05 indicated a significant difference. Student’s *t*-test was also used to analyze the significant differences between indicated groups and control.

## Supplementary Materials

The following are available online, Figure S1: Effects of different concentrations of cadmium on the growth of the wheat seedlings. Figure S2: Effects of different concentrations of cadmium treatment on the wheat seed germination. Figure S3: Effects of exogenous hydrogen peroxide treatment on the growth of wheat seedlings. Figure S4: Cadmium induced leaf senescence in the wheat seedlings. Figure S5: Expression of the melatonin biosynthesis genes in response to senescence treatment. Figure S6: Effects of auxin treatment on the root growth. Figure S7: Effects of auxin on the seedling growth under cadmium stress. Table S1: List of primer sequences used in the experiments.
