# Supplementary material for: Exogenous Melatonin Confers Cadmium Tolerance by Counterbalancing the Hydrogen Peroxide Homeostasis in Wheat Seedlings
Source: Molecules. 2018 Mar 30;23(4):799. doi: 10.3390/molecules23040799 (PMC6017192; doi:10.3390/molecules23040799)
Supplement: Supplementary file 1 [file molecules-23-00799-s001.pdf]

Supplementary information

Exogenous melatonin confers cadmium tolerance by counterbalancing the hydrogen peroxide homeostasis in wheat seedlings

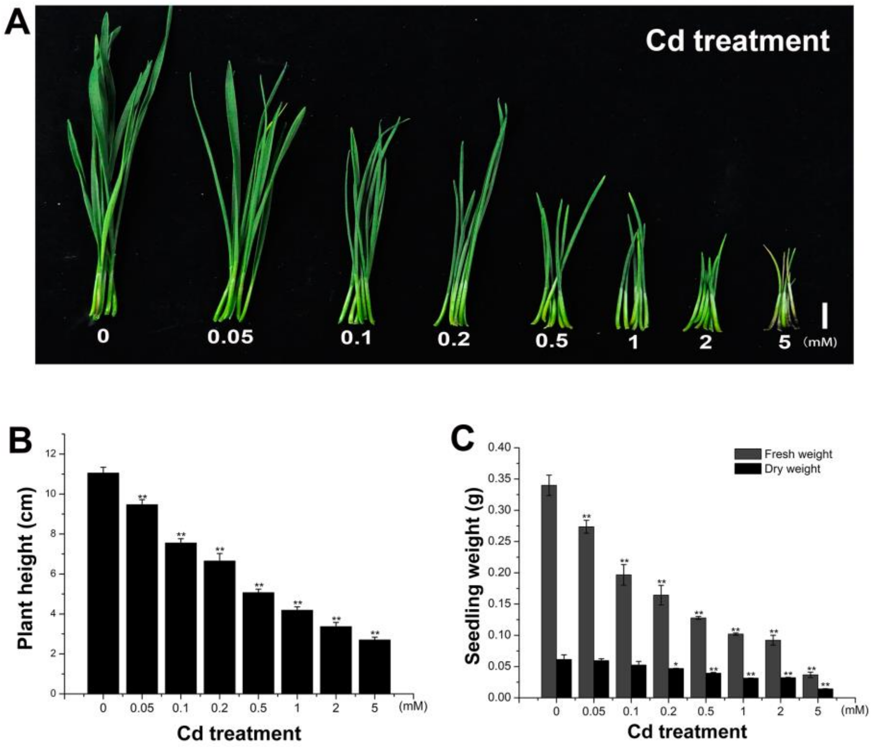

Figure S1. Effects of different concentrations of cadmium on the growth of the wheat seedlings. Three-day-old wheat seedlings were root-treated with different concentrations of cadmium **(A)**. Plant height **(B)**, fresh and dry weight **(C)** of the shoots were calculated one week after cadmium treatment (n = 20). For determination of plant fresh and dry weight, three seedlings were mixed as one sample. **V**alues are mean±SE. Significance between treatment and control was determined by Student's t test. Significance level: $^{**}P < 0.01$, $^{*}P < 0.05$. Bars = =1 cm.

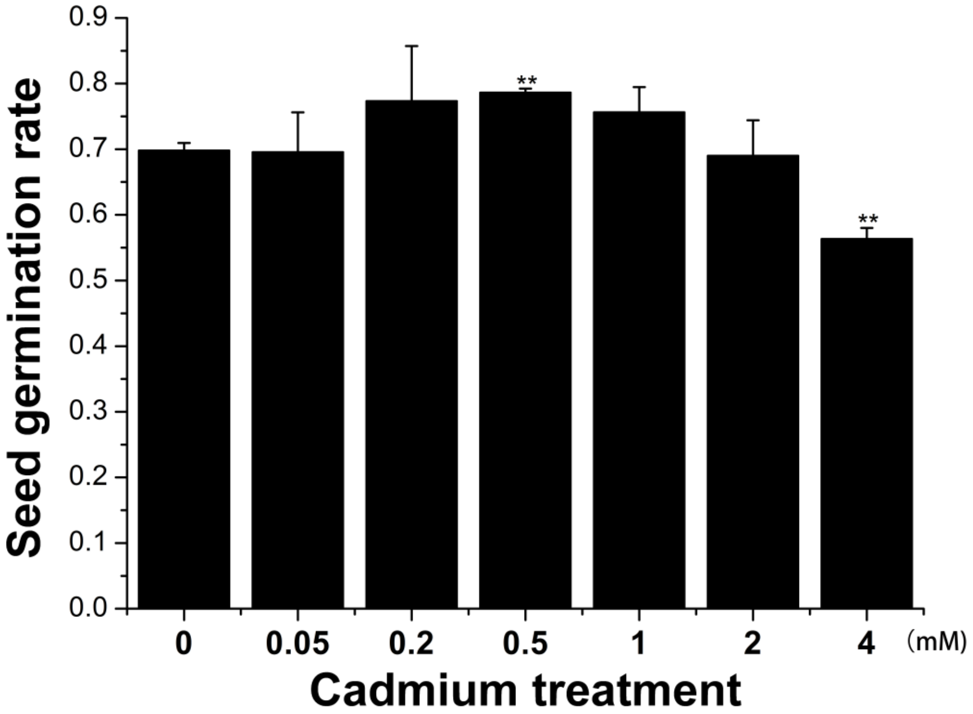

Figure S2. Effects of different concentrations of cadmium treatment on the wheat seed germination. The germination rate was recorded at 24 h after seed priming with different concentrations of cadmium (n = 4). **V**alues are mean±SE. Significance between indicated groups and control was determined by Student's t test. Significance level: $^{**}P < 0.01$.

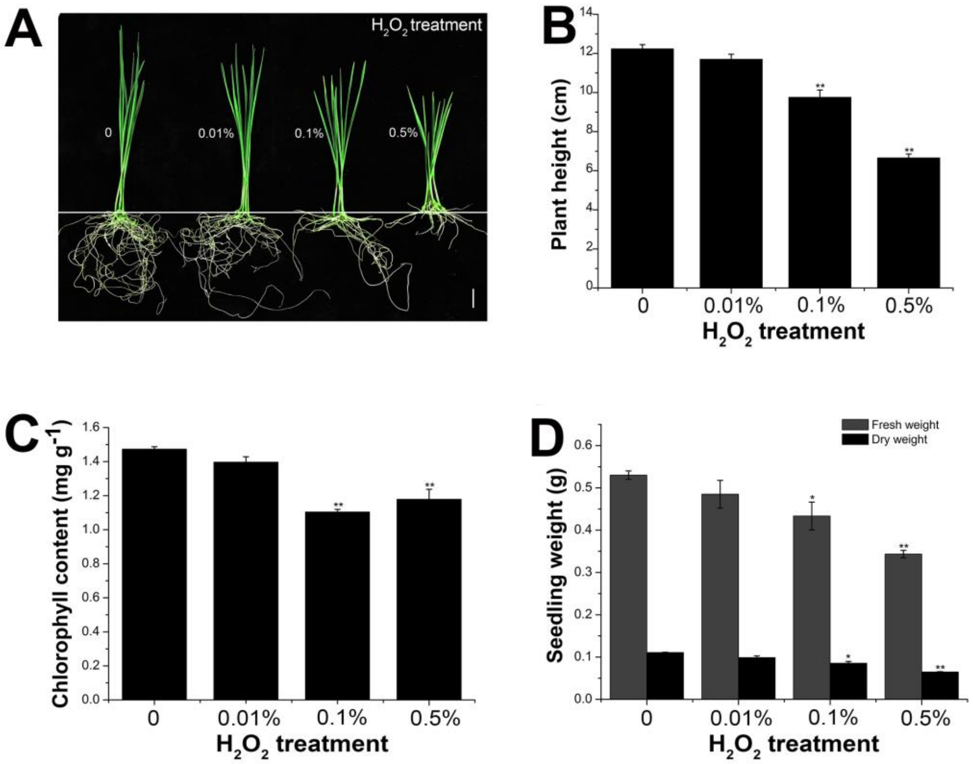

Figure S3. Effects of exogenous hydrogen peroxide treatment on the growth of wheat seedlings. (A) Phenotypes of wheat seedlings, one week after root treatment with H_2O_2. Plant height **(B)**, total chlorophyll content **(C)** and seedling weight **(D)** were separately calculated after exogenous H_2O_2 treatments.

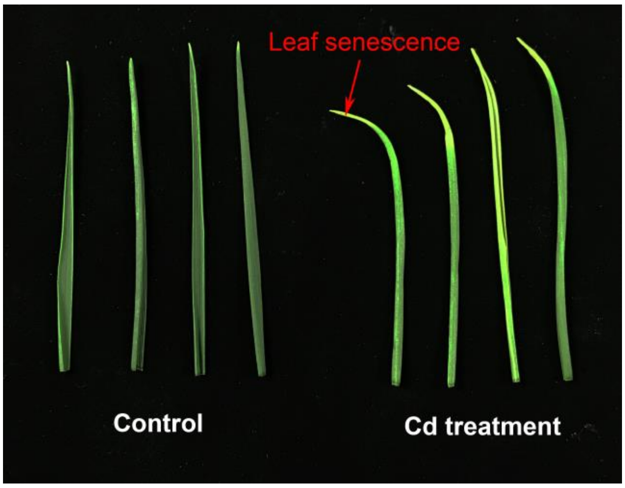

Figure S4. Cadmium induced leaf senescence in the wheat seedlings. 10 days after root treatment with water or 0.2 mM cadmium on the one-week-old wheat seedlings growing in the petri dish, the leaves were taken for photograph. The red arrow indicated the senescence of the leaf tip.

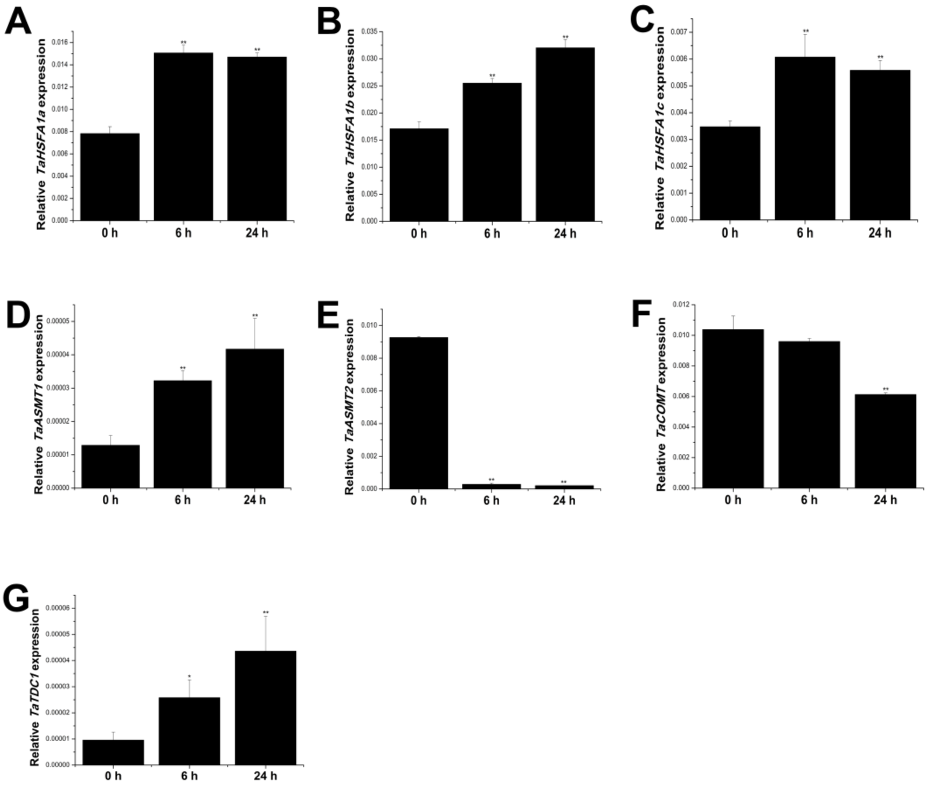

Figure S5. Expression of the melatonin biosynthesis genes in response to senescence treatment. The expression of *TaHSFA1a* **(A)**, *TaHSFA1b* **(B)**, *TaHSFA1c* **(C)**, *TaASMT1* **(D)**, *TaASMT2* **(E)**, *TaCOMT* **(F)** and *TaTDC1* **(G)** were analyzed at 0 h, 6 h and 24 h after senescence treatment using qPCR (n = 3). *TaACT* was used as the internal reference. **V**alues are mean ± SE. Significance between indicated groups and control was determined by Student's t test. Significance level: $^{**}P < 0.01$, $^{*}P < 0.05$.

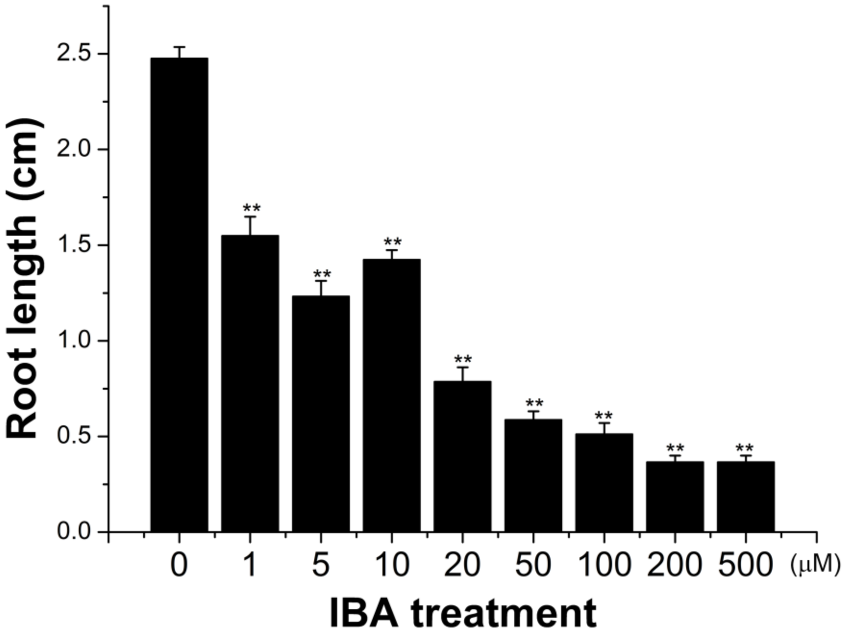

Figure S6. Effects of auxin treatment on the root growth. Root length was measured at 2 d after root treatment with different concentrations of IBA (n = 20). **V**alues are mean ± SE. Significance between indicated groups and control was determined by Student's t test. Significance level: $^{**}P < 0.01$.

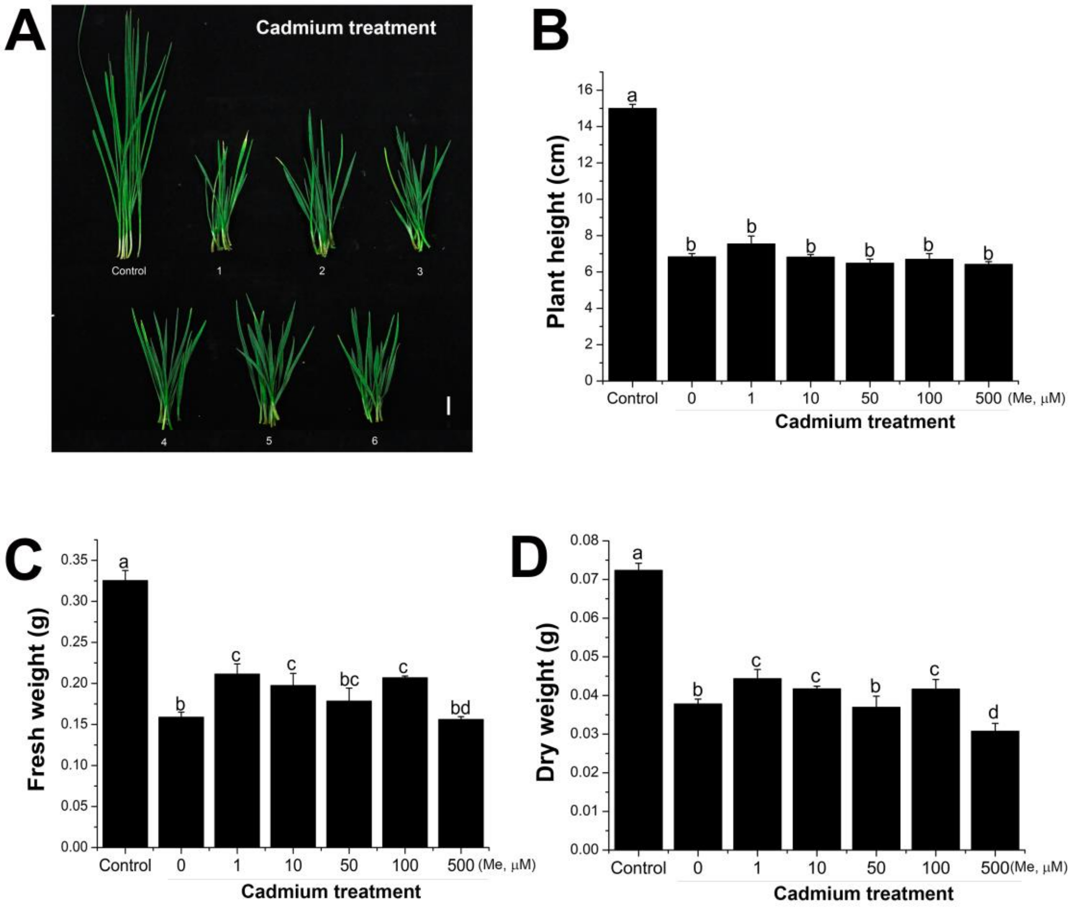

Figure S7. Effects of auxin on the seedling growth under cadmium stress. (A) Photograph of representative leaves in response to different concentrations of IBA treatment under cadmium stress. Plant height **(B)**, fresh weight **(C)** and dry weight **(D)** were calculated one week after treatment (n = 20). **V**alues are mean ± SE. Means with different letters are significantly different at $P < 0.05$ using Tukey's test.

Table S1 List of primer sequences used in the experiments.

Gene name	Full name	GenBank ID	Direction	Primer sequences
TaACT	*ACTIN*	KX533928.1	Forward	GTATCGTGTTGGATTCTGGTGATGGT
			Reverse	CGGCAGTGGTGGTGAAGGAGTA
TaHSFA1a	*Heat shock transcription factor A1a*	KF208541.1	Forward	ATGCTCCAATGCCTTCTTGTGTAGAG
			Reverse	GCTGCTGCCGTTGCTCCATT
TaHSFA1b	*Heat shock transcription factor A1b*	KF208542.1	Forward	TACCAGATGAAGGCAGTGATGACTCT
			Reverse	ACCTGAATCCACCTCGTCGTTGT
TaHSFA1c	*Heat shock transcription factor A1c*	KF208543.1	Forward	CATCTACTGAAGACCATCAACAGAAGGAA
			Reverse	TCTACACAAGAAGGCATTGGAGCATT
TaASMT1	*N-acetylserotonin methyltransferase*	HG670306.1	Forward	GGCAGAGTGATCGTGATGGACCT
			Reverse	CCAGGATCTTGTAGCCGCTGAATC
TaASMT2	*N-acetylserotonin methyltransferase*	HG670306.1	Forward	CAACCTTATGGATAGACCTCGATGACAC
			Reverse	GCTGATGGATGTGGCAATGATGGTA
TaCOMT	*Caffeic acid O-methyltransferase*	EF413031.1	Forward	GGGCAGAAGCAAGCAGAGATGG
			Reverse	CGATGGCGTTCTTCAGCGTCAT
TaTDC1	*Tryptophan decarboxylase*	GU817319.1	Forward	CGTGCCGAGAAACTTTGCTTTGG
			Reverse	GCCTCTCCTCCTGAAGTGACGAT
